# Velocity mapping of the aortic flow at 9.4 T in healthy mice and mice with induced heart failure using time-resolved three-dimensional phase-contrast MRI (4D PC MRI)

**DOI:** 10.1007/s10334-014-0466-z

**Published:** 2014-11-08

**Authors:** Philipp Rene Bovenkamp, Tobias Brix, Florian Lindemann, Richard Holtmeier, Desiree Abdurrachim, Michael T. Kuhlmann, Gustav J. Strijkers, Jörg Stypmann, Klaus H. Hinrichs, Verena Hoerr

**Affiliations:** 1 Department of Clinical Radiology, University Hospital Münster, Albert-Schweitzer-Campus 1, Building A16, 48149 Münster, Germany; 2 Department of Computer Science, University of Münster, Münster, Germany; 3 Department of Cardiology and Angiology, University Hospital Münster, Münster, Germany; 4 Biomedical NMR, Department of Biomedical Engineering, Eindhoven University of Technology, Eindhoven, The Netherlands; 5 Center for Imaging Research and Education (CIRE), Eindhoven, The Netherlands; 6 European Institute for Molecular Imaging (EIMI), Münster, Germany; 7 Department of Biomedical Engineering and Physics, Academic Medical Center, Amsterdam, The Netherlands

**Keywords:** Small-animal imaging, Blood flow velocity, MRI, Cardiovascular system

## Abstract

**Objectives:**

In this study, we established and validated a time-resolved three-dimensional phase-contrast magnetic resonance imaging method (4D PC MRI) on a 9.4 T small-animal MRI system. Herein we present the feasibility of 4D PC MRI in terms of qualitative and quantitative flow pattern analysis in mice with transverse aortic constriction (TAC).

**Materials and methods:**

4D PC FLASH images of a flow phantom and mouse heart were acquired at 9.4 T using a four-point phase-encoding scheme. The method was compared with slice-selective PC FLASH and ultrasound using Bland–Altman analysis. Advanced 3D streamlines were visualized utilizing Voreen volume-rendering software.

**Results:**

In vitro, 4D PC MRI flow profiles showed the transition between laminar and turbulent flow with increasing velocities. In vivo, 4D PC MRI data of the ascending aorta and the pulmonary artery were confirmed by ultrasound, resulting in linear regressions of *R* ^2^ > 0.93. Magnitude- and direction-encoded streamlines differed substantially pre- and post-TAC surgery.

**Conclusions:**

4D PC MRI is a feasible tool for in vivo velocity measurements on high-field small-animal scanners. Similar to clinical measurement, this method provides a complete spatially and temporally resolved dataset of the murine cardiovascular blood flow and allows for three-dimensional flow pattern analysis.

**Electronic supplementary material:**

The online version of this article (doi:10.1007/s10334-014-0466-z) contains supplementary material, which is available to authorized users.

## Introduction

Pathologies of the heart and cardiovascular system are among the major diseases of our time. As such, powerful investigative methods for the detection, characterization, and even prevention of cardiac disease are needed both in the clinic and in preclinical studies.

Quantitative blood flow analysis of the cardiovascular system is a popular approach for gaining insight into the physiological and pathological mechanisms of the heart [1]. In addition to ultrasound (US), phase-contrast magnetic resonance imaging (PC MRI) has been recognized in clinical studies in recent years for velocity mapping of cardiovascular blood flow [2, 3]. Time-resolved slice-selective PC MRI techniques have been shown to provide reliable velocity maps to aid in the diagnosis of myocardial infarction [4], liver function [5] and multiple sclerosis [6]. With further advancement, time-resolved phase-contrast techniques combined with three-dimensional volume acquisition—the so-called 4D PC MRI methods—have been developed to investigate the cardiovascular system in three-dimensional velocity vector graphs [7–11]. Both slice-selective PC MRI and optical methods have been used to verify the accuracy of 4D flow velocity measurements [11, 12]. In three-dimensional volume imaging, the whole heart is covered in a single scan, and additional information can be obtained regarding neighboring voxels [7–13]. The temporal evolution of blood flow velocity is measured and assessed quantitatively in different vessels, which allows for a detailed description and picture of cardiac and valvular functionality. Both the absolute velocity values and flow pattern characteristics provide important quantitative data in the diagnosis of aortic aneurysms [14] or aortic valve prosthesis function [10]. Streamlines visualized as tangential traces to three-dimensional velocity fields are especially powerful for the analysis of different flow patterns [8, 9].

Over the last few years, advanced genetic engineering has led to a variety of transgenic cardiovascular mouse models, including apolipoprotein E (ApoE) knockout, scavenger receptor class B type I (SR-B1) knockout, and ApoE/SR-B1 double-knockout (hypoE) [15–17]. These models develop spontaneous and long-term atherosclerotic lesions and myocardial infarction, which have already been characterized in the literature [18]. In addition, sophisticated surgical animal models of heart failure, such as transverse aortic constriction (TAC)-induced cardiac hypertrophy, have also been developed [19, 20], and are usually validated by blood flow measurements [21, 22].

While slice-selective PC MRI is a standard tool for preclinical studies [22–26], the application of 4D PC MRI on small-animal MRI systems is still challenging. As high technical requirements are necessary, the approaches that have been developed thus far for three-dimensional PC MRI in small animals are all based on multi-slice acquisition techniques [27, 28]. These techniques do not benefit from volume excitation and improved spatial resolution. Due to the very small structures and the rapid cardiac cycles of mice, fast-responding gradient systems are needed for 4D PC MRI, and long scan times are required as well. Even fast 4D PC MRI techniques in clinical studies suffer from lengthy measurement durations [8]. In order to obtain a comprehensive quantitative disease description and to assess the influence of local flow perturbation on the global hemodynamic system, a complete picture of blood flow circulation is necessary, including information about predominant flow direction and 3D flow incoherencies such as vortices or helical patterns.

The aim of this work was to establish a time-resolved three-dimensional acquisition method, including three-dimensional velocity encoding, for detailed qualitative and quantitative analysis of cardiovascular blood flow in a mouse heart on a small-animal MR system at 9.4 T. We validated our 4D flow method, both in vitro and in vivo, with gold-standard techniques such as slice-selective PC MRI and US [21, 29–31], and studied hemodynamic flow patterns in healthy mice and mice with TAC [20, 32]. As preliminary work for future quantitative studies of cardiovascular diseases in mouse models, we customized the Voreen volume-rendering software [33] to apply streamline visualization to 4D PC MRI data, allowing different blood flow patterns to be distinguished.

## Materials and methods

### Study overview and workflow

**Fig. 1:**
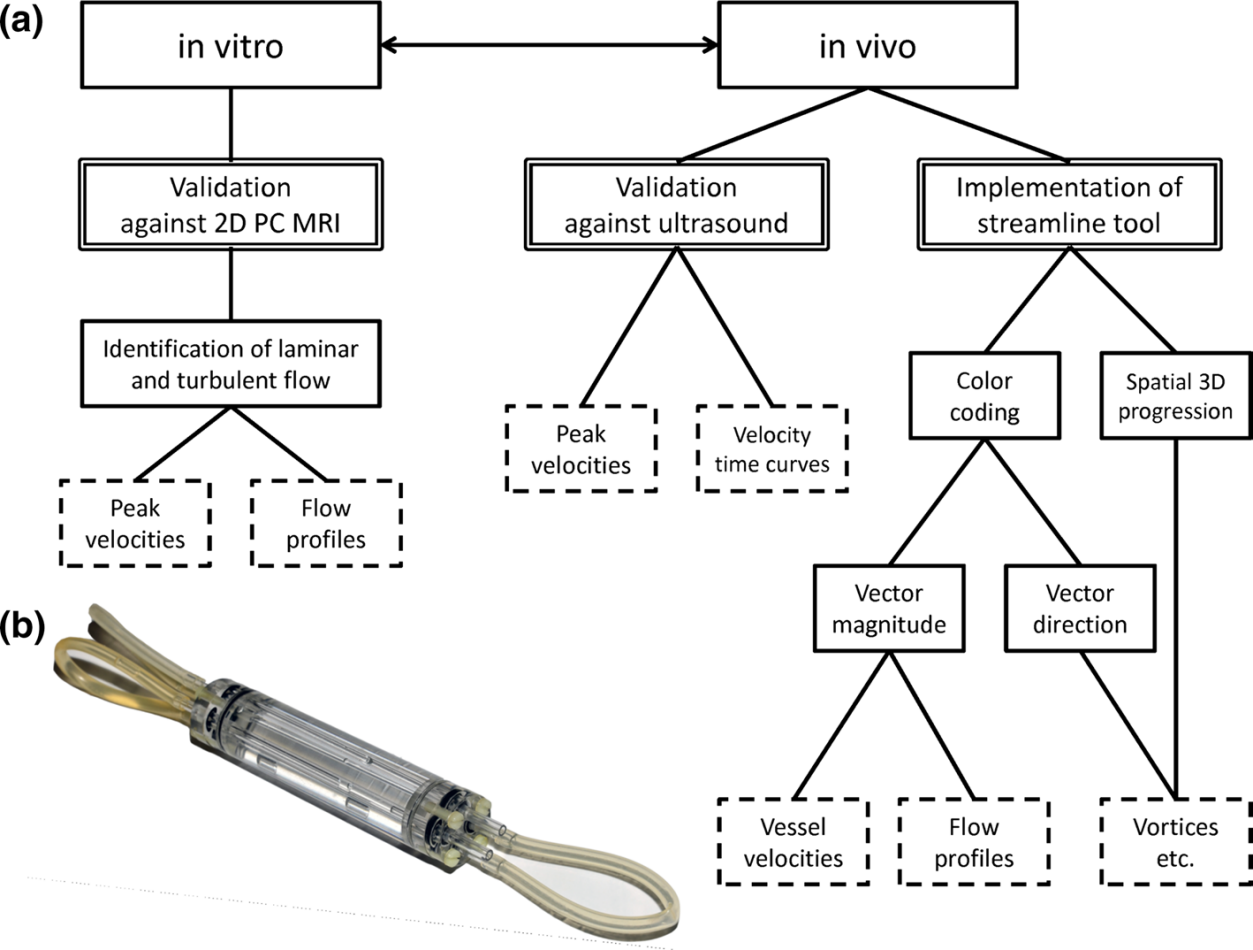
**a** Workflow diagram of the present study: first, validation of 4D PC MRI in vitro against slice-selective 2D PC MRI in a flow phantom, which enables the identification of laminar and turbulent flows inside the phantom from peak velocities and reconstructed flow profiles; second, validation of the method in vivo in mice against the gold-standard ultrasound in terms of peak flow velocity quantification and velocity–time curve reconstruction; third, implementation of a streamline analysis tool for high-quality visualization and improved analysis of flow behaviors. Color encoding for either vector magnitude or vector direction representation provides information regarding vessel velocities and flow profiles as well as structures in the blood flow such as vortices or helical streams. **b** Photograph of the flow phantom, comprising a water-filled acryl glass body with four parallel tubes (internal diameter: 3 mm), which are connected to each other in series and attached to an adjustable peristaltic pump

The study presented herein is divided into three parts: first, in vitro validation of 4D PC MRI; second, in vivo validation of 4D PC MRI in mice, including the application on an exemplary mouse model of cardiovascular hypertrophy; and third, implementation of a streamline visualization tool. Figure 1a provides a detailed flowchart of the validation steps. In the first step, the 4D PC MRI method was validated in vitro against slice-selective 2D PC MRI in order to verify that the transition from slice to volume acquisition, which occurs in tandem with changes in spatial resolution, did not systematically affect the accuracy of the measurements.

In the second step, the 4D PC MRI method was validated in mice against the gold-standard ultrasound method in order to demonstrate the feasibility of in vivo measurements. In the last step, post-processing analysis was performed on the 4D PC MRI data to calculate streamlines using Voreen volume-rendering software. The spatial distribution and orientation of streamlines provided deep insight into murine blood flow dynamics and behavior.

### In vitro flow model

The design of the flow phantom (Fig. 1b) comprises a water-filled cylindrical acrylic glass body (diameter 30 mm) with four tubes (diameter 3 mm) placed inside. The four tubes are connected in series and attached to a peristaltic pump (Medorex e.K., Hörten-Hardenberg, Germany). The peristaltic pump has a variable pumping rate of *Q* = 0–800 ml/min, enabling adjustment of water flow velocities inside the tubes in a range of *v* = 0–340 cm/s. The flow phantom covers variable steady-flowing water inside the tubes as well as static water inside the acrylic glass body surrounding the tubes. This arrangement improves SNR and shimming due to the high amount of MR-sensitive medium and allows for the observation of scan quality derived from small variations in the velocity values of the static water.

### Mouse models

All animal procedures were conducted in accordance with the German animal protection laws and were approved by the animal care committee of the local government (Az. 84-02.04.2013.A046, 87-51.04.2011.A003; North Rhine-Westphalia State Agency for Nature, Environment and Consumer Protection).

#### Validation experiments

Measurements were performed on a group of seven healthy female C57BL/6 mice. Animals approximately 12 weeks of age were measured with both US and MRI. During the measurements, the mice were kept anesthetized by inhaled anesthesia consisting of a 0.7/0.3 air/O_2_ mixture and 1.5–2.5 % isoflurane. Throughout the duration of the MRI procedure (approximately1.5 hours), the mouse hearts were kept at a mean cardiac period of 123–150 ms, depending on the mouse. The corresponding electrocardiograms (ECG) showed only small variations (0.8–5.8 %) over the measurement time.

#### Transverse aortic constriction (TAC)

In two male and two female ApoE-R61^h/h^ mice, pressure overload-induced cardiac hypertrophy and heart failure was induced by TAC, as previously described [34]. In brief, under deep anesthesia (0.04 mg fentanyl/4 mg midazolam/kg body weight, combined with 1.5 % v/v isoflurane/O_2_), the thorax was opened and a surgical thread placed around the aortic arch between the brachiocephalic artery and the left common carotid artery. By occluding the aorta with a 27-gauge blunted needle inserted into the suture loop and subsequent removal of the needle, the aorta was then constricted to a defined diameter. The aortic constriction then generated pressure overload in the left ventricle, which ultimately led to non-ischemic dilated cardiomyopathy.

To assess the affects of the TAC surgery, the blood velocities in the left and right carotid arteries (LCA, RCA) were measured by MRI one week before (baseline) and one week after TAC surgery. During MRI experiments, the ApoE-R61^h/h^ mice were kept anesthetized in the same manner as the C57BL/6 mice in the validation experiments. The mean cardiac period was 137–146 ms, with small variations in a range of 2.0–3.5 %.

### MRI

#### Measurements

In vivo MRI was performed on a 9.4 T high-field small-animal MRI system with a horizontal bore of 20 cm and ParaVision 5.1 operation software (BioSpec 94/20 USR, Bruker BioSpin, Ettlingen, Germany). For data acquisition, a 35-mm-diameter mouse body quadrature volume coil (Rapid Biomedical, Rimpar, Germany) was used in a 1-T/m gradient insert (BGA-6S, Bruker BioSpin, Ettlingen, Germany).

After anesthesia with isoflurane was administered, the mice were connected to a small-animal monitoring unit (SA Instruments, Inc., Stony Brook, NY, USA) comprising ECG and respiration monitoring, and were positioned inside the magnet. For positioning of the velocity-encoded FOV, a self-gated cine FLASH sequence was used (TR/TE: 5.1/2.6 ms, FA: 15°, FOV: 25 × 25 mm^2^, slice thickness: 1 mm, MTX: 256 × 256). Blood flow measurements were performed using a three-dimensional ECG- and respiration-triggered FLASH sequence with bipolar gradients for flow velocity encoding (Flowmap, Bruker BioSpin, Ettlingen, Germany). Applying a four-point Hadamard encoding scheme, all three spatial velocity components were encoded simultaneously [35, 36]. The sequence parameters were TE/TR: 2.2/6.9 ms, FA: 15°, FOV: 15 × 28 × 20 mm^3^, MTX: 200 × 100 × 30, NA: 3, resulting in an absolute scan time of approximately one hour when the ECG and respiration triggering was taken into account (14 time frames averaged about 30,000 cardiac cycles; in vitro scan time: 4 min).

For validation purpose, an ECG- and respiration-triggered slice-selective 2D FLASH sequence with a four-point Hadamard encoding scheme for flow velocity encoding was used (Flowmap, Bruker BioSpin, Ettlingen, Germany). The sequence parameters were TE/TR: 2.8/15.0 ms, FA: 30°, FOV: 30 × 30 mm^2^, slice thickness = 1 mm, MTX: 256 × 256, NA: 10, resulting in an absolute scan time of 2.6 minutes in vitro and 30–40 minutes in vivo (7–9 time frames).

#### Data analysis

FID files of the PC MRI datasets were imported into MATLAB (The MathWorks, Inc., Natick, MA, USA) for reconstruction. Magnitude and phase images were separated from the complex image data. The magnitude image was used to filter the phase images and cut off the statistical phase noise in low-signal regions. Velocity vectors were assembled from the three velocity components and were investigated in regions of high flow. The peak velocity for different vessels was obtained by determining the length of the velocity vectors inside the vessels. Substantial changes in the flow velocities induced by TAC surgery were shown graphically.

### Ultrasound

US measurements were performed on a Vevo 2100 ultrasound system (FUJIFILM Visualsonics, Toronto, Canada) at 32 MHz using a high-resolution transducer for vascular imaging in mice. After real-time acquisition of the pulse wave velocity, the peak velocity of the blood flow was calculated and averaged over 15–20 cardiac cycles. Statistical differences between US and 4D PC MRI measurements were evaluated with Bland–Altman analysis [37] in SigmaPlot (Systat Software, Inc. [SSI], San Jose, CA, USA).

### Streamline visualization

To quantitatively visualize the time-resolved three-dimensional velocity MRI data, three-dimensional streamlines (instantaneous traces tangent to the velocity field for each time frame) were calculated. To this end, reconstructed data were exported from MATLAB into Voreen (Münster University, Visualization and Computer Graphics Research Group, Münster, Germany; [33]). For streamline rendering, a region of interest (ROI) covering the whole heart and great vessels was selected. In this ROI, the velocity data of random seed voxels were compared with neighboring voxels for each time frame individually. This streamline calculation is based on line integral convolution (LIC) using a fourth-order Runge–Kutta integration algorithm, as previously described [38]. To improve the flexibility and quality of the reconstructed streamline images, the streamline rendering was controlled by parameters describing the total number of maximally created streamlines, the minimal length of each streamline, and the minimal and maximal vector magnitude threshold values. In this streamline reconstruction tool, two different color-encoding schemes are employed. The first represents the absolute velocity by vector magnitude encoding in a hot metal color scale, while the second is a vector direction encoding in a rainbow color. Movies of temporal streamline evolution were generated by combining streamline graphs of all time frames.

From streamline images, different flow patterns were analyzed to distinguish between vortices, turbulences, and helical, stable, or unstable flow. Vortices are characterized by localized twists of streamlines leading to reduced blood flow propagation. Helical flow also shows twists; however, these still follow the predominant direction of the vessel. In contrast, turbulences are much more difficult to illustrate in streamlines. Because of sudden changes in flow direction in turbulent regions, turbulent streamlines are usually short and discontinuous.

## Results

### Validation

#### Flow velocities and profiles in vitro

**Fig. 2:**
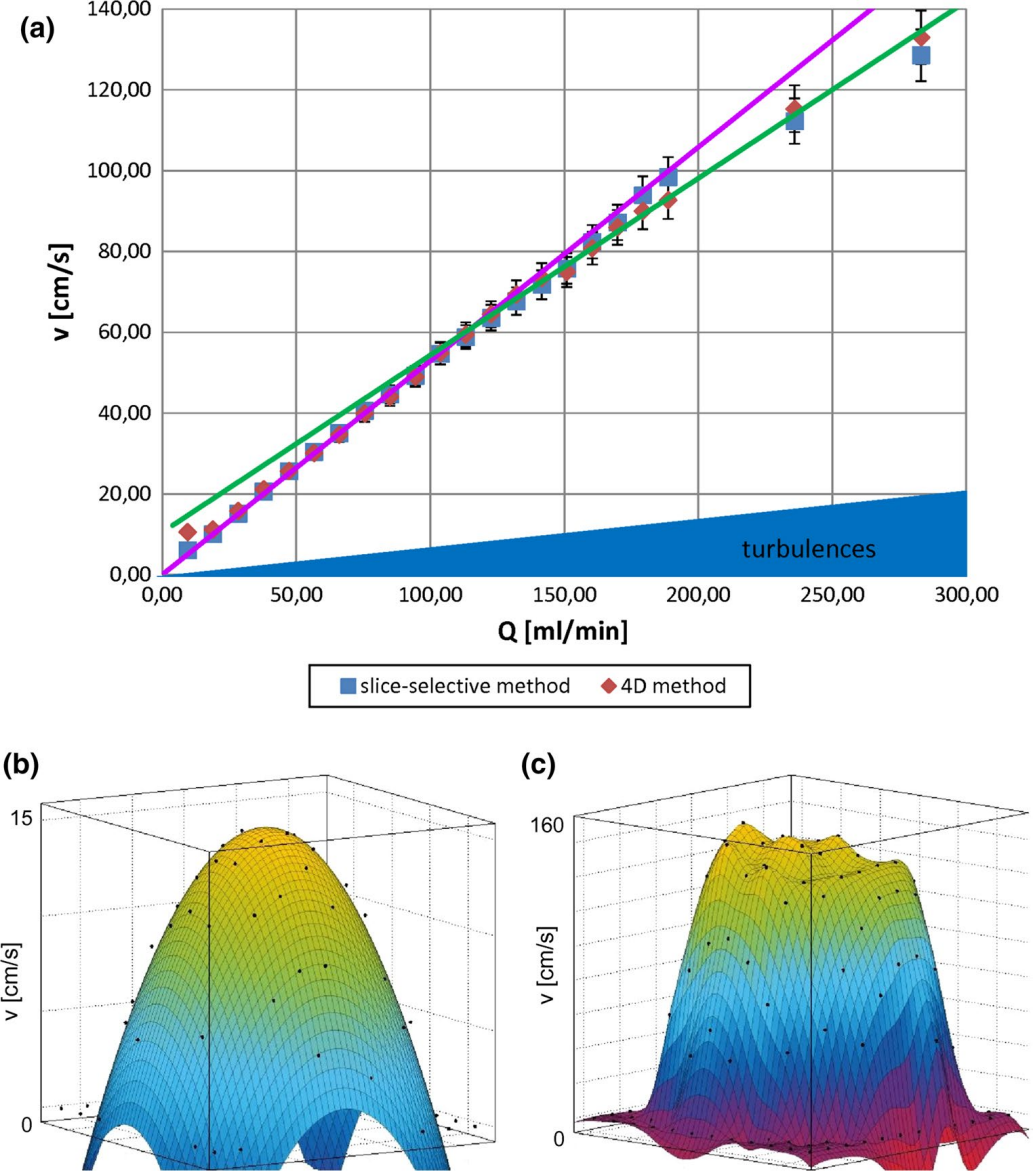
In vitro validation of 4D PC FLASH MRI. **a** Validation of 4D PC FLASH MRI (*diamonds*) against an established time-resolved slice-selective 2D PC FLASH method (*squares*): velocity *v* (cm/s) is shown for different pumping rates *Q* (ml/min) in the flow phantom (see Fig. 1b). The results are averaged over three measurements. Both datasets show a bend at about 115 ml/min, which is illustrated by two linear regressions, for low (0, 100 ml/min) (*pink line*) and high pumping rates (100, 300 ml/min) (*green line*). The bend indicates a phase transition from laminar to turbulent flow behavior, wherein the flow is laminar for pumping rates up to about 100 ml/min. A transition state from laminar to turbulent flow can be observed for higher pumping rates and is noticeable in the dampened increase in flow velocity. **b** and **c** show the corresponding spatial flow profiles of in vitro 4D PC FLASH MRI data obtained for different pumping rates. **b** Laminar flow profile for a low pumping rate (*Q* = 30 ml/min) and low peak velocity with two-dimensional parabolic fit (*R* ^2^ = 0.9424). **c** Turbulent flow profile for a high pumping rate (*Q* = 300 ml/min) and high peak velocity characterized by parabolic boundaries and a plateau in the center of the flow profile

To evaluate the accuracy of flow calculations based on 4D PC FLASH data, 4D velocity measurements (SNR = 58.5 ± 3.7, 3 averages, *v*_max_ = 120 cm/s) were validated against an established slice-selective 2D PC method (SNR = 61.6 ± 2.9, 10 averages, *v*_max_ = 120 cm/s) for different velocities on a flow phantom (see “Materials and methods”, Fig. 1b). Figure 2 shows the velocities obtained with each method. Excellent agreement is found for the peak velocities. The velocity course clearly demonstrates the transition between laminar and turbulent flow at the different velocities examined. To illustrate that the velocities and flow profiles follow different physical principles at different velocity ranges, the data are linearly fitted by two linear regressions for the range of low (0, 100 ml/min; *R* ^2^ = 0.9778) and high pumping rates (100, 300 ml/min; *R* ^2^ = 0.9977). The two lines cross each other at a transition area (at a pumping rate around *Q* = 115.7 ml/min) where flow profiles change from laminar to turbulent.

Figure 2b, c shows flow profiles for different pumping rates calculated from 4D PC MRI datasets. For low pumping rates—represented by a maximum velocity of *v*_max_ = 15 cm/s—the flow velocity is low and laminar. As shown in Fig. 2b, laminar flow data is characterized by a parabolic flow velocity profile and is in good agreement with a parabolic fit (*R* ^2^ = 0.9424). Figure 2c represents the flow profile at a high pumping rate—represented by a *v*_max_ = 160 cm/s—of the same flow phantom. Compared to the laminar (and parabolic) flow, the flow profile is flatter, and the parabolic fit deviates substantially from the measured values (*R* ^2^ = 0.8219).

#### Flow velocities and velocity–time curves in vivo

**Fig. 3:**
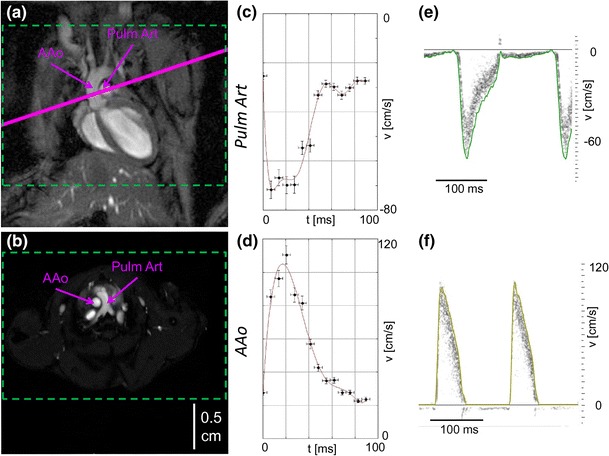
In vivo validation of 4D PC FLASH MRI in mice. **a**, **b** Anatomical overview obtained with self-gated FLASH showing images of the mouse heart in coronal (**a**) and axial view (**b**), wherein the *solid purple line* in (**a**) corresponds to the selected slice in (**b**) in the slice-selective 2D PC MRI experiments. For 4D PC MRI experiments, a three-dimensional FOV covering the whole heart and great vessels was chosen (*green dashed lines*). **b** illustrates the locations in which the velocities are calculated in the ascending aorta (AAo) and the pulmonary artery (Pulm Art). **c**–**f** present the temporal evolution of peak flow velocities in velocity–time curves for the two vessels [Pulm Art (**c**, **e**) and AAo (**d**, **f**)] for 4D PC MRI (**c**, **d**) and ultrasound (**e**, **f**). The *green* and *yellow lines* in **e** and **f** are the envelopes of the ultrasound velocity data.

To assess the accuracy and feasibility of the 4D PC FLASH technique in vivo, blood flow measurements were performed with both MRI and US. The maximum blood flow velocities for two cardiac vessels, the pulmonary artery (Pulm Art) and the ascending aorta (AAo) were measured for healthy C57BL/6 mice at the age of 12 weeks (Fig. 3a, b). Temporal evolution of the maximum velocity is given in velocity–time curves for the Pulm Art (Fig. 3c, e) and the AAo (Fig. 3d, f) with both MRI (Fig. 3c, d) and US measurements (Fig. 3e, f). The plots show the same qualitative and quantitative evolution for both modalities, but originate from different acquisition principles. While the US dataset is real-time-resolved for each single cardiac cycle, the data points obtained by PC MRI are averaged over the total measurement time, which corresponds to approximately 30,000 cardiac cycles. Under isoflurane narcosis, the mice showed a stable cardiac period in the range of 123–150 ms, depending on the mouse.

**Fig. 4:**
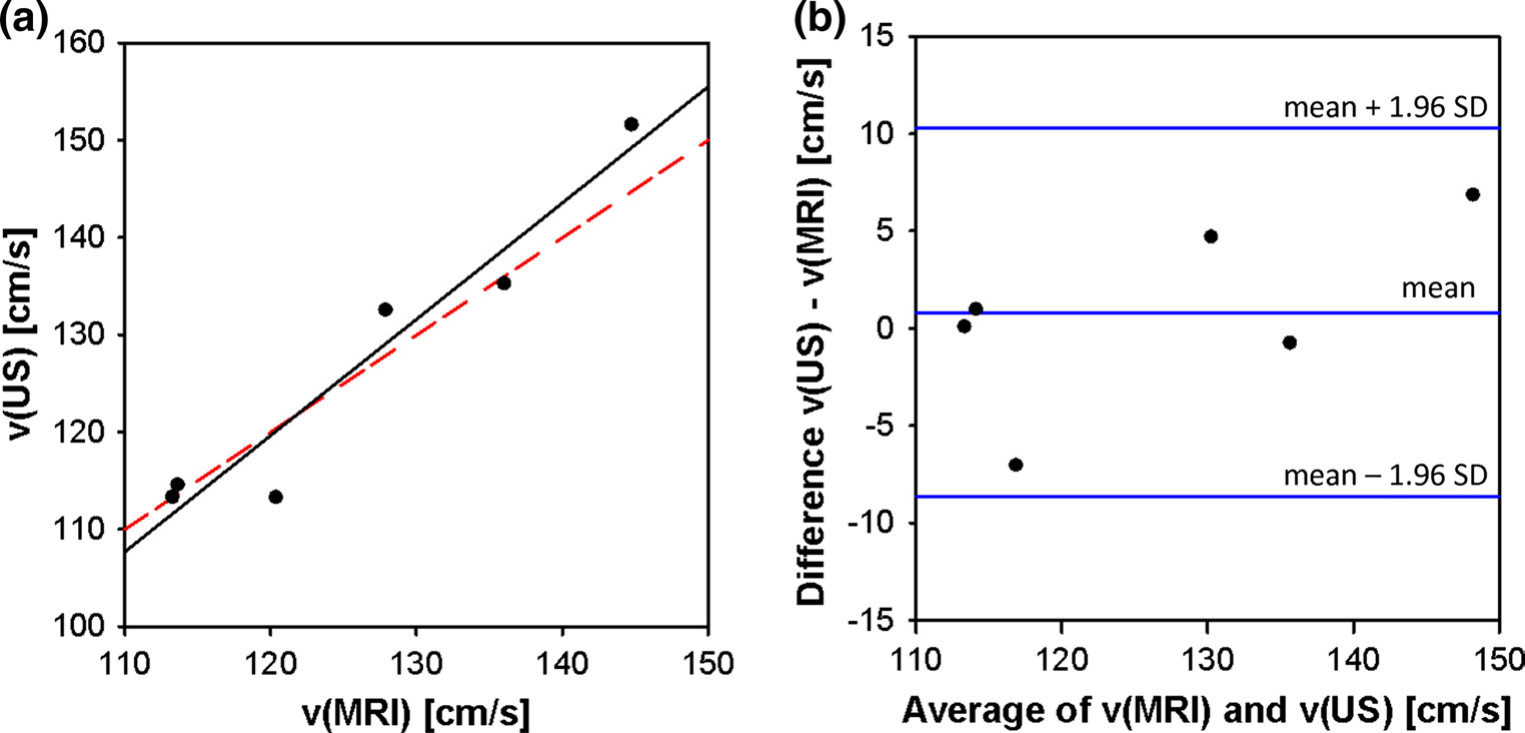
Bland–Altman statistics for method validation of 4D PC MRI against ultrasound, obtained in vivo in mice for the ascending aorta. **a** Plot of peak flow velocities obtained by ultrasound and by 4D PC MRI showing linear regression (*black line*; *R* ^2^ = 0.9304) and line of identity (*red dashed line*). **b** Bland–Altman plot for method comparison: all observed data points are included in narrow limits of the interval amplitude, indicating good agreement of both methods.

In Table 1, the quantitative results of the 4D PC MRI and the US measurements are summarized as mean values with standard deviations. Corresponding Bland–Altman analysis was performed and is shown for the AAo in Fig. 4. The values from both methods show good agreement, resulting in linear regressions with *R* ^2^ = 0.9304 for the AAo and *R* ^2^ = 0.9687 for the Pulm Art. The Bland–Altman plot (Fig. 4b) includes all observed data points and shows quite narrow limits of the interval amplitude, indicating good agreement of 4D PC MRI and US.

**Table 1:** Peak flow velocities of the pulmonary artery (Pulm Art) and the ascending aorta (AAo) in healthy C57BL/6 mice obtained with 4D PC MRI and Doppler ultrasound (US)

	Pulm Art		AAo	
	*v* (cm/s)	*N*	*v* (cm/s)	*N*
4D PC MRI	74 ± 27	7	126 ± 11	7
US	71 ± 6	6	127 ± 14	6

Data is presented as mean ± SD

### Quantitative flow analysis in a TAC mouse model

**Fig. 5:**
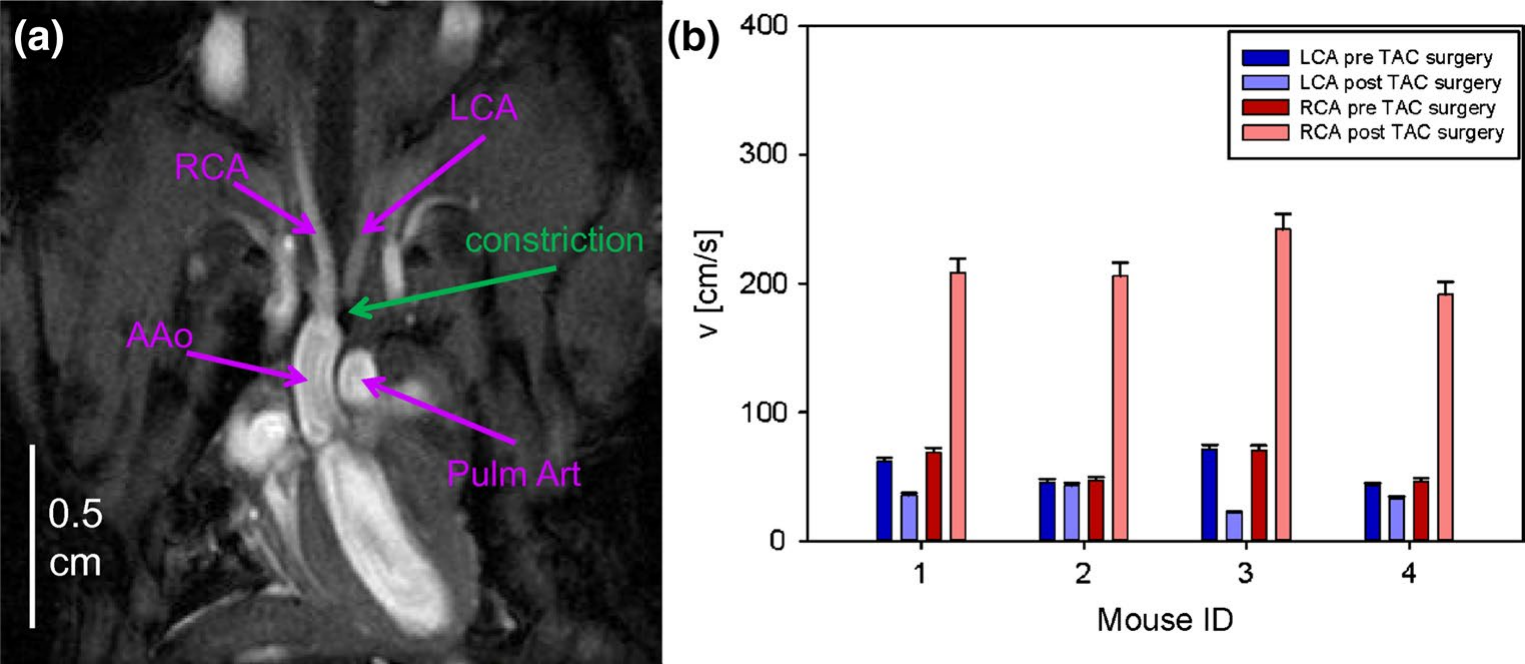
Transverse aortic constriction (TAC) study. **a** Anatomical overview obtained with self-gated FLASH in a coronal view showing the ascending aorta (AAo), the pulmonary artery (Pulm Art), and the carotid arteries (left and right carotid artery [LCA, RCA]), as well as the constriction of the aortic arch. **b** Bar chart of the peak flow velocities in the LCA and RCA before and one week after TAC surgery. The peak flow velocity in the RCA, in particular, shows a substantial difference between the pre- and post-surgery measurements.

In preclinical small-animal studies, the constriction of the aortic arch is a common experimental procedure used to induce cardiac hypertrophy [20, 32]. In order to characterize cardiac failure, peak flow velocities in the AAo, Pulm Art, RCA, and LCA were investigated in ApoE-R61^h/h^ mice, before and one week after TAC surgery, by 4D PC MRI and slice-selective 2D PC MRI, respectively (Fig. 5a). The results are shown in Tables 2 and 3. Peak velocities are substantially different after surgery. While velocity values are lower in the AAo, the Pulm Art, and the LCA, the flow velocity in the RCA has increased.

**Table 2:** Peak blood flow velocity of the pulmonary artery (Pulm Art) and the ascending aorta (AAo) in ApoE-R61^h/h^ mice obtained by 4D PC MRI before and one week after transverse aortic constriction (TAC) surgery

	Pulm Art		AAo	
	*v* (cm/s)	*N*	*v* (cm/s)	*N*
Before surgery	288 ± 20	3	370 ± 10	3
After surgery	97 ± 10	4	150 ± 7	4

Data is presented as mean ± SD

**Table 3:** Peak blood flow velocities of the right and left carotid arteries (RCA, LCA) in ApoE-R61^h/h^ mice obtained by slice-selective 2D PC MRI before and one week after transverse aortic constriction (TAC) surgery

	RCA		LCA	
	*v* (cm/s)	*N*	*v* (cm/s)	*N*
Before surgery	58 ± 12	4	56 ± 12	4
After surgery	212 ± 19	4	34 ± 7	4

Data is presented as mean ± SD.

### Streamline analysis

To demonstrate the full power of 4D PC MRI in quantitative flow pattern analysis, we calculated streamlines using the Voreen image processing and three-dimensional rendering software tool. In Voreen, streamlines are rendered from the three-dimensional velocity data and are visualized time-resolved in three dimensions on an exemplary slice of the reconstructed three-dimensional magnitude image.

**Fig. 6:**
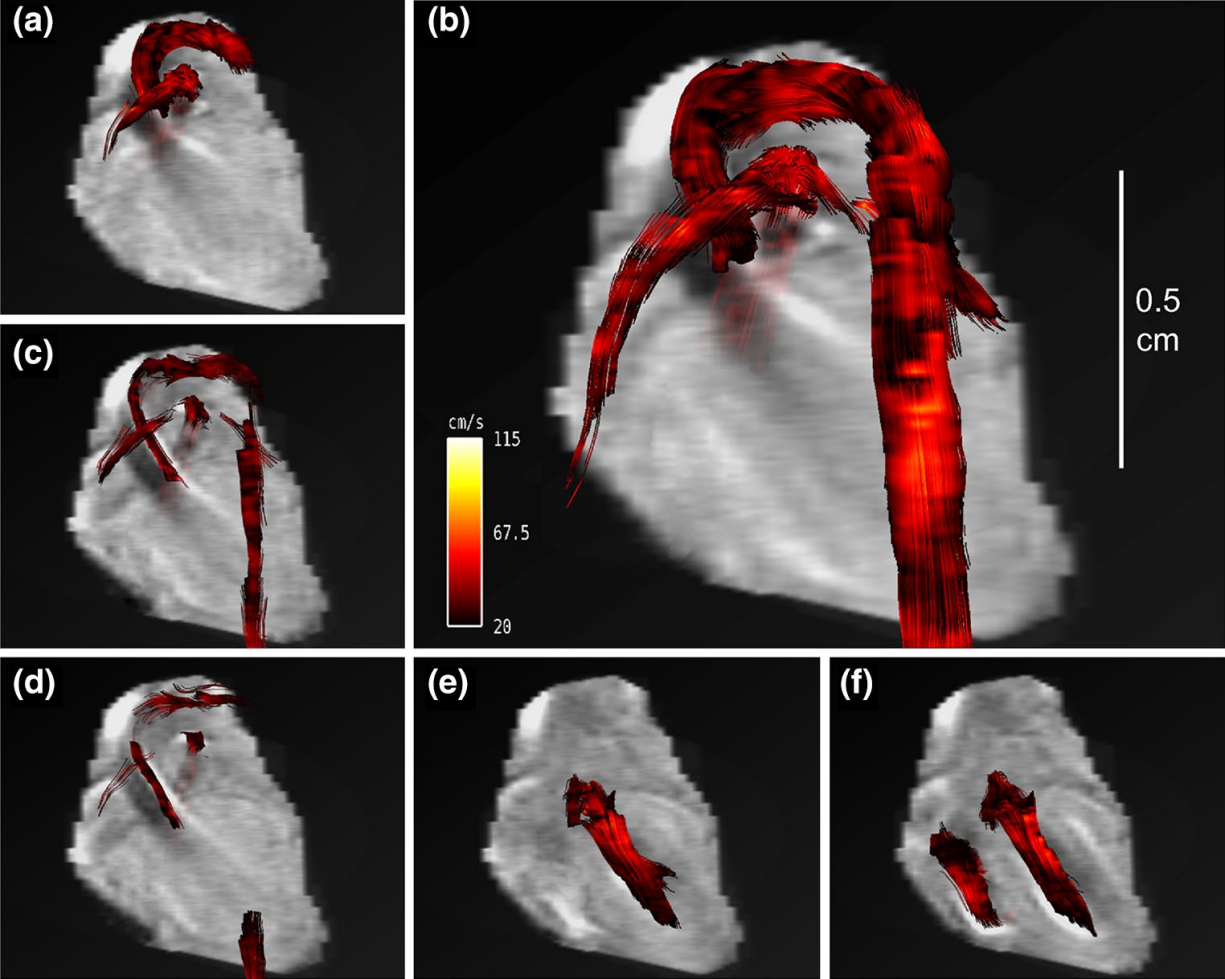
Blood flow visualization with vector magnitude-encoded streamlines in the cardiovascular system of a mouse at selected time frames: **a** 6.88 ms, **b** 13.76 ms, **c** 41.28 ms, **d** 48.16 ms, **e** 68.80 ms, and **f** 75.68 ms after R wave of the ECG. **a**–**d** Represent the blood flow through the aortic arch and the pulmonary trunk in the systole. In the late systole (**c**, **d**) the streamlines become shorter and collapse. **e**, **f** Blood inflow into the left ventricle during the diastolic phase. A movie of the vector magnitude-encoded streamline data is provided in Online Resource 1.

**Fig. 7:**
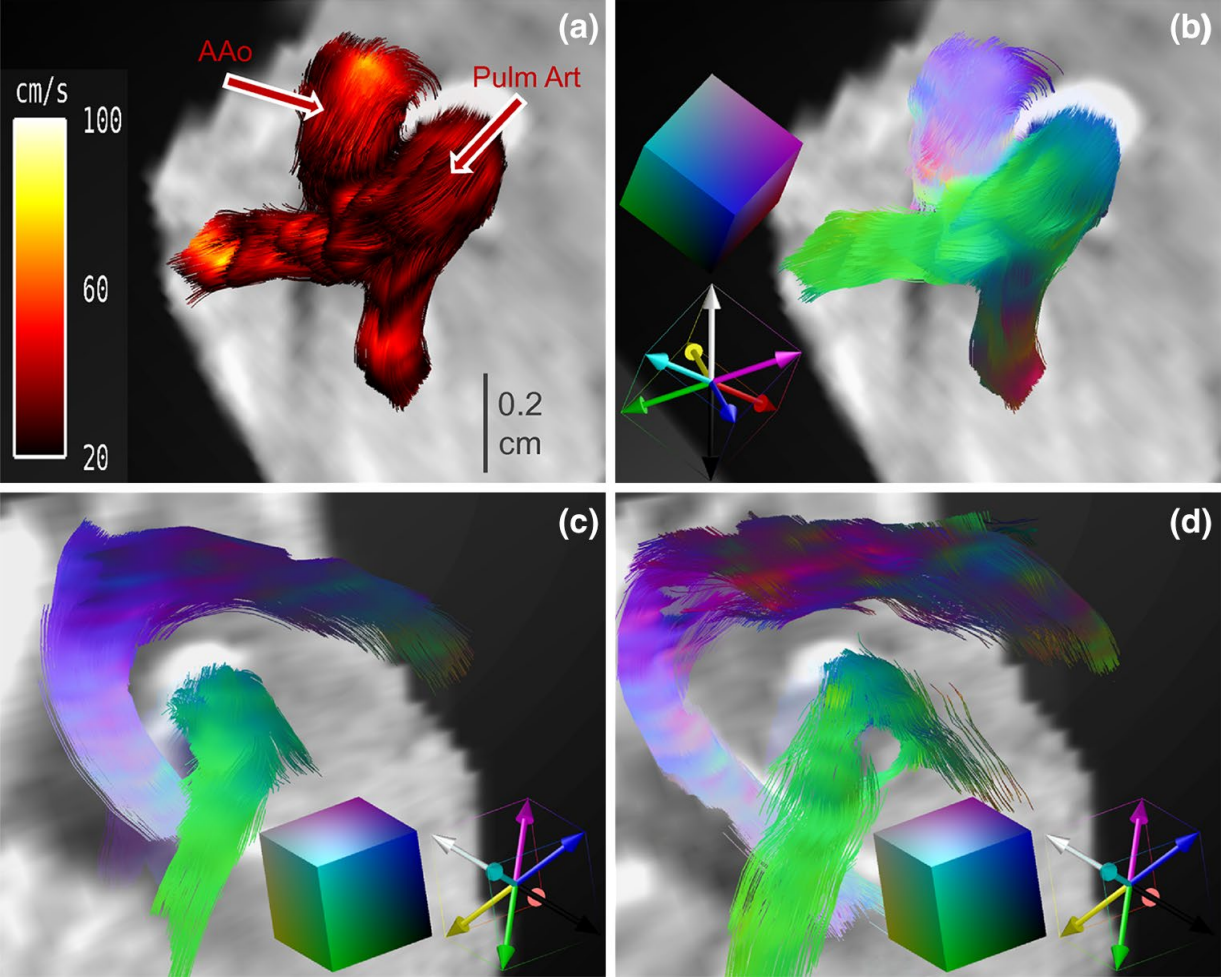
Streamline analysis. **a** Zoom of the vector magnitude-encoded streamlines represented in Fig. 6a for a smaller region of interest (ROI) showing streamlines of the ascending aorta (AAo) and the pulmonary trunk. In this view, it can be clearly seen that the color coding in the cross section of the AAo represents a laminar flow profile, with highest velocity in the vessel center. **b** Zoom as in (**a**) with vector direction encoding: the encoding scheme is shown in two overlays; first, a cube showing the colors for the directions heading towards the observer, and second, diagonals of the same cube with colored cube edges for illustration. **c**, **d** Vector direction-encoded streamlines for a zoom into the coronal view given in Fig. 6a, c. **c** Coherent streamlines in the early systole. **d** Streamlines in late systole begin to twist helically, which can be nicely visualized with the large number of colors present, representing numerous flow directions. A movie of the vector direction-encoded streamline data is provided in Online Resource 2.

From an in vivo 4D PC MRI dataset covering the entire mouse heart, streamlines for selected time frames are shown in Fig. 6a–f. Among the different time frames, the evolution of blood flowing through the ascending aorta, aortic arch, descending aorta, and pulmonary artery, in addition to the inflow of blood in the left ventricle, is visualized. A cine movie is provided in Online Resource 1. Predominant flow velocities are illustrated in a hot metal color scale. In the early systole (Fig. 6a), the blood flow profile in the cross section of the ascending aorta has a parabolic shape, showing highest velocity values in the vessel center (Fig. 7a). In addition to the vector magnitude encoding in the hot metal color scale, the streamline tool provides vector direction encoding. In this second color-coding scheme, the tangential direction of the streamline points is shown in rainbow colors (Fig. 7b). For this color coding, the diagonals of a cube are given predefined colors, leading to mixed colors for the remaining directions. In Fig. 7b, the color coding is illustrated with two overlays. The first is a cube showing the complete spectrum of mixed colors for the directions that process towards the observer. The second overlay shows the color coding for the diagonals and edges of the cube to illustrate the spatial distribution of the color encoding.

In Fig. 7c, d, the direction-encoded streamlines are presented in two time frames of the systole: first, the early systole in Fig. 7c, and second, the late systole in Fig. 7d; the time frames correspond to the vector magnitude-encoded images in Fig. 6a, c. In the early systole, the flow pattern in the ascending aorta is described by coherent streamlines. This can be seen very distinctly in the vector direction encoding, as all streamlines possess the same color (Fig. 7c). In contrast, in the late systole, the streamlines in the aortic arch begin to twist. The color encoding of the streamlines exhibits a large variety of colors, from bluish to reddish. This clearly shows that the streamlines twist about 180°, indicating a helical flow pattern (Fig. 7d). A cine movie is provided in Online Resource 2. Finally, when all blood is pumped out of the left ventricle, the flow is decreased, and the streamlines through the aortic arch are intermittent (as shown in vector magnitude encoding in Fig. 6d).

**Fig. 8:**
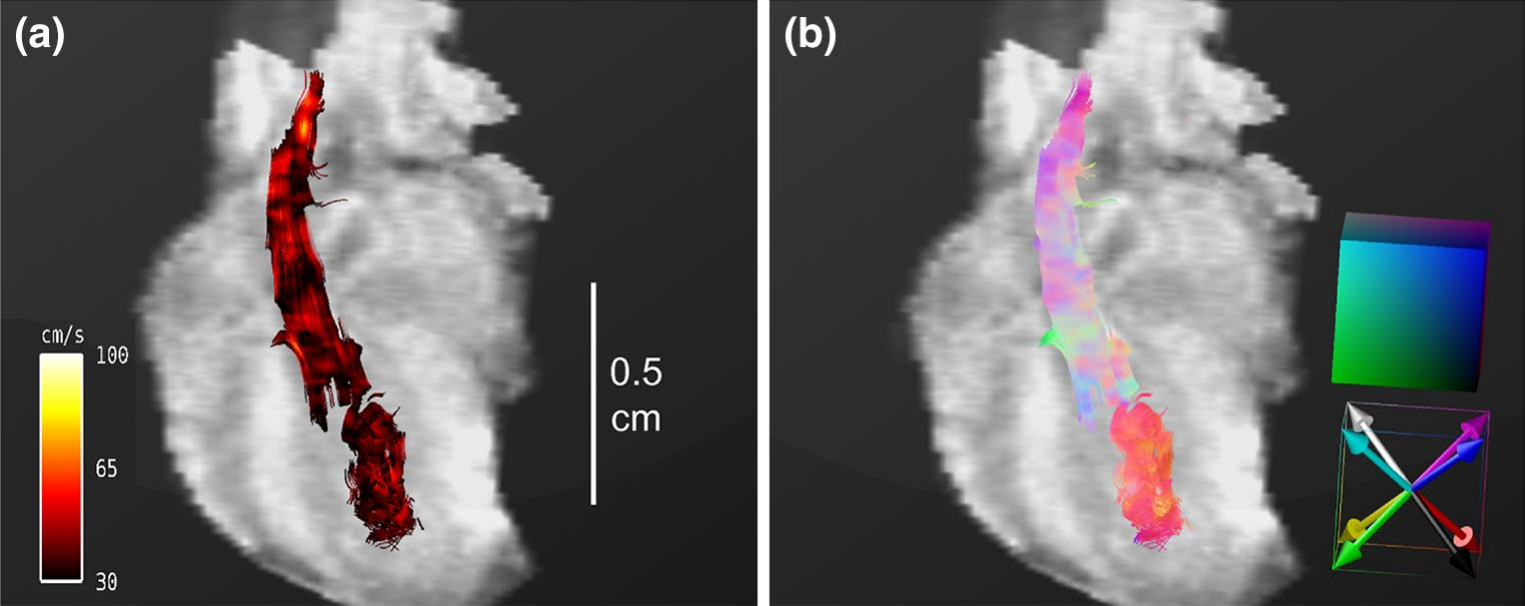
Streamline analysis of mice with transverse aortic constriction (TAC). **a** Vector magnitude-encoded streamlines for the cardiovascular system at 6.88 ms after R wave of the ECG in a coronal view, as shown in Fig. 6. **b** Vector direction-encoded streamlines of the same ROI and time point. Due to the constriction, the predominant velocity direction does not follow the aortic arch, but is cranial, following the branches of the right carotid artery.

In Fig. 8, corresponding streamlines after TAC surgery are visualized in vector magnitude (Fig. 8a) and vector direction encoding (Fig. 8b) in the early systole. The streamlines show predominant cranial blood flow through the RCA during the systole, while streamlines of the blood flow through the aortic arch are not visible. In the vector direction encoding, the aortic streamlines show mainly white and pink colors, indicating cranial flow. Additionally, residual flow in the heart is visible in downward-tending streamlines (reddish color in the ventricle and the tip of the heart.

## Discussion

The potential of PC MRI for quantitative clinical and preclinical blood flow investigations has been recognized in previous research [2–6]. In clinical studies, 4D PC MRI has provided impressive results on human MR scanners [7–11, 13], while slice-selective 2D PC MRI techniques are still commonly used in preclinical studies [23, 24]. Therefore, it was our aim to establish a 4D PC MRI method on a high-field small-animal MR scanner, allowing us to obtain a complete and detailed picture of the cardiovascular flow and hemodynamic system in a mouse model of cardiac hypertrophy.

Flow measurements with our established 4D PC FLASH method, both in vitro and in vivo (Figs. 2, 3, 4; Table 1), showed good agreement with the results obtained by slice-selective 2D PC MRI and US, which confirmed the accuracy of the 4D method on a small-animal scanner.

The 4D dataset allowed for individual adjustment of the spatial resolution in all three dimensions, limited only by scan time and gradient performance. As no time-consuming slice-selection gradients are applied in the 4D technique, a shorter TE was also achieved. Additional gain in SNR in three-dimensional images further improved image quality, as well as flow quantification, due to reduced noise in the phase-difference images. Three rather than 10 averages, therefore, were needed to obtain the same SNR in the 4D method compared to the slice-selective method.

Using a flow phantom, we showed that both slice-selective and 4D PC MRI provided detailed characterization and identification of the predominant flow. For different pumping rates, different flow profiles were obtained. While the flowing water showed a laminar flow profile for low pumping rates and low Reynold’s numbers, as velocities increased, the flow profile deviated from the parabolic profile into a transition state consisting of an intermediate between laminar and turbulent flow (Fig. 2b, c).

The calculation of flow profiles is not a special application of 4D PC MRI. However, by three-dimensional acquisition, flow profiles can be calculated for freely selected locations and orientations within any vessel of interest. In contrast, in slice-selective PC MRI methods, this calculation is limited to a preselected cross section of a defined vessel. This difference renders the 4D PC MRI technique superior to other multi-slice PC approaches, especially as these methods often suffer from large inhomogeneities in spatial resolution [27, 28] due to limited gradient performance, which may lead to partial volume effects and underestimation of flow velocities for vessels that are small compared to the spatial resolution [39].

In vivo experiments comparing 4D PC MRI and US also illustrated the advantages of the MRI technique. Accurate information regarding flow characteristics was obtained for both the Pulm Art and the AAo in a single 4D PC FLASH scan. In contrast, US measurements depend on the relative direction between blood flow and the ultrasonic beam, and must be performed for each vessel independently [40]. The US technique claimed the highest accuracy for the Pulm Art because of the nicely matching relative orientation of the vessel direction and the high sensitivity of the ultrasonic transducer.

Phase-contrast velocity data are typically visualized as greyscale or color-coded phase images of the velocity components, as introduced by Bryant et al. [41] and van Dijk [42]. A drawback of this visualization technique, however, is that it requires an image for each velocity component. Furthermore, only one or the other of velocity direction or value can be displayed. When the length of the velocity vector is illustrated in a single color-coded view, the information about the flow direction is lost. Finally, for a volume acquisition dataset, cross sections of the data must be selected for reconstruction of the images.

Novel visualization techniques were introduced for human 4D PC MRI datasets by Markl et al. [8]. Additional information about surrounding voxels obtained by the 4D technique was used for streamline visualization. Our in vivo experiments showed that streamline rendering is also possible in the tiny cardiovascular systems of the mouse heart. Streamlines provide information about both the velocity direction and the magnitude. In Figs. 6 and 7 and the Online Resource 1, the flow through the aortic arch is illustrated by magnitude-encoded streamlines for different time frames. The bending of the aortic arch is clearly visualized, and shows the highest velocity in the center of the vessel. Additional information about changes in the flow direction is provided by vector direction encoding in Fig. 7c, d and Online Resource 2. Compared to human studies in the cine mode, due to the high murine heart rate and the finite temporal resolution, some streamlines vanished when the cine traversed from one time frame to another. However, as with previous human studies [8, 9], we obtained information regarding different flow patterns, including helical flow, vortices, turbulences, and unstable flow. While the AAo showed coherent streamlines, the streamlines in the aortic arch began to twist, and the flow pattern was helical. In contrast, the streamlines after TAC surgery did not follow the direction of the aortic arch and did not pass the constriction (see Fig. 8). Because of the pressure overload in the aorta, blood flow in both the AAo and the Pulm Art decreased substantially. In addition, the blood flow velocity in the LCA was also reduced, while the velocity in the RCA was increased. These results are in good agreement with previous US studies by Hartley [32]. Streamline visualization revealed that the predominant velocity direction was cranial and followed the branch of the RCA. Coherent streamlines from the AAo to the right carotid were without vortices or twists, indicating a straight vascular geometry.

The study of flow patterns plays an important role in revealing cardiac function. For example, vortices supply the appropriate momentum for the redirection of the blood flow in the ventricles, thereby improving ejection efficiency [43, 44]. As these patterns are sensitive to any changes in the vascular diameter, flexibility, and curvature, they also provide a better understanding of animal disease models.

## Conclusion

In conclusion, we established a 4D PC MRI method for blood flow analysis of the cardiovascular system of the mouse heart. To the best of our knowledge, this is the first in vivo study demonstrating the feasibility of 4D PC MRI with streamline visualization on a high-field small-animal MRI scanner. Our results showed good quantitative accuracy compared with slice-selective 2D PC MRI and Doppler US measurements. In contrast to multi-slice PC MRI, often published as 4D PC MRI methods, our 4D PC MRI method benefits from the features of volume acquisition in terms of shorter TE and improved resolution.

We have shown that the major advantage of small-animal 4D PC MRI derives from its post-processing potential. Using the Voreen image processing and three-dimensional rendering software tool, we were able to study flow patterns in multiple vessels from high-dimensional MRI datasets. The characterization and identification of helical torsion or vortices in these patterns was also made possible. The strength of our 4D PC MRI methodology was demonstrated by comparing the blood flow behavior in healthy C57BL/6 mice to mice with heart failure induced by TAC surgery. A single measurement with the 4D PC MRI protocol allowed for the investigation of quantitative peak flow velocities in multiple vessels over the whole cardiac cycle, providing the corresponding flow pattern evolution as well as the predominant flow directions. Thus, 4D PC MRI proved to be a valuable tool for the analysis of the entire hemodynamic physiology of small-animal disease models in preclinical studies.

## Electronic supplementary material

Below is the link to the electronic supplementary material.

Movie of 4D PC MRI vector magnitude-encoded streamline data reconstructed from a healthy mouse heart (C57BL/6), as described in Fig. 6. The movie shows the blood flow streamline evolution of the ascending aorta, the aortic arch, the descending aorta, and the pulmonary artery in a complete cardiac cycle (AVI 1007 kb).

Movie of 4D PC MRI vector direction-encoded streamline data reconstructed from a healthy mouse heart (C57BL/6), as described in Fig. 7c/d. The movie shows the blood flow streamline evolution of the ascending aorta, the aortic arch, the descending aorta, and the pulmonary artery in the systole (AVI 1189 kb).

## Abbreviations

AAo: Ascending aorta
ApoE: Apolipoprotein E
ECG: Electrocardiogram
FA: Flip-angle
FID: Free induction decay
FLASH: Fast low-angle shot
FOV: Field of view
hypoE: Hypomorphic apolipoprotein E
KO: Knockout
LCA: Left carotid artery
LIC: Line integral convolution
MTX: Matrix size
MRI: Magnetic resonance imaging
NA: Number of averages
PC: Phase-contrast
Pulm Art: Pulmonary artery
RCA: Right carotid artery
ROI: Region of interest
SNR: Signal-to-noise ratio
SR-B1: Scavenger receptor class B type I
TAC: Transverse aortic constriction
TE: Echo time
TR: Repetition time
US: Ultrasound
